# Myocardial strain measured via two-dimensional speckle-tracking echocardiography in a family diagnosed with arrhythmogenic left ventricular cardiomyopathy

**DOI:** 10.1186/s12947-021-00270-1

**Published:** 2021-12-20

**Authors:** Changsheng Ma, Jiali Fan, Bingyuan Zhou, Caiming Zhao, Xin Zhao, Bo Su, Yuzhu Miao, Yuping Liao, Li Wang

**Affiliations:** The First Affiliated Hospital of Soochow University, Suzhou, China

**Keywords:** Arrhythmogenic left ventricular cardiomyopathy, Two-dimensional speckle-tracking echocardiography, Layer-specific strain, Left ventricular longitudinal strain

## Abstract

**Background:**

Arrhythmogenic cardiomyopathy is a myocardial disorder characterized by ventricular arrhythmias, right and/or left ventricular involvement, and fibrofatty infiltrations in the myocardium. We report a family diagnosed with arrhythmogenic left ventricular cardiomyopathy (ALVC) and depict their echocardiographic characteristics.

**Methods and results:**

Fifteen family members were divided into three groups based on whether they carried the TMEM43 mutation and had been diagnosed with ALVC. Eight of them had TMEM43 mutations, and four were diagnosed with ALVC according to the Padua criteria. Only the proband experienced sudden cardiac death and had a dilated left ventricle. Left ventricular ejection fraction was reduced in two patients; however, left ventricular global longitudinal strain was depressed in three patients. Low QRS voltages in limb leads were evident in three patients, and five patients had frequent ventricular premature contractions. Late gadolinium enhancement was evident in three patients. Left ventricular layer-specific strain showed that the transmural strain gradient ratio was increased in patients diagnosed with ALVC, and it was elevated in the genotype-positive and phenotype-negative groups compared with healthy individuals.

**Conclusion:**

Global left ventricular longitudinal strain better evaluated left ventricular function than left ventricular ejection fraction. The transmural strain gradient ratio was elevated in patients diagnosed with ALVC, suggesting that it was useful for the evaluation of ALVC.

## Introduction

An expert panel of the Heart Rhythm Society (HRS) defined arrhythmogenic cardiomyopathy (ACM) as an arrhythmogenic disorder of the myocardium that is not explained by ischemia, hypertensive, or valvular disease [1]. Arrhythmogenic right ventricular cardiomyopathy (ARVC) is a classic phenotype characterized by fibrofatty myocardial replacement in the right ventricle. Recent studies have shown that myocardial scarring is also evident in the left ventricle or biventricle [2, 3]. Arrhythmogenic left ventricular cardiomyopathy (ALVC) is characterized by left ventricular involvement predominantly with no or minor RV abnormalities [4]. Several studies have provided significant insights into the diagnosis of ALVC based on contrast-enhanced cardiac magnetic resonance (CE-CMR) tissue characterization findings, depolarization/repolarization ECG abnormalities, and ventricular arrhythmia features for the diagnosis of the LV phenotype [4, 5]; however, the echocardiographic characteristics of ALVC have not been well described. We report a family with a TMEM43 mutation diagnosed with ALVC and emphasize the characterization of left ventricular longitudinal strain in this family, with the aim of providing an advanced understanding of ALVC.

## Methods

### Population and data collection

The study was approved by the local research ethics committee, and all individuals provided written informed consent. Fifteen pedigree members were included in this study, and four of them were diagnosed with ALVC at the First Affiliated Hospital of Soochow University. Clinical evaluation included 12-lead ECG, 24-h Holter, echocardiography and contrast-enhanced cardiac magnetic resonance (CE-CMR). Clinical manifestations, disease history, and family history of the pedigree members were also collected.

Diagnosis was based on the Padua criteria for ALVC. These criteria were developed by a multidisciplinary team of basic researchers and clinical cardiologists at the Medical School of the University of Padua by taking into account the tissue characterization findings by contrast-enhanced cardiac magnetic resonance, depolarization/repolarization ECG abnormalities, and ventricular arrhythmia features for the diagnosis of the left ventricular phenotype, which are grouped into six categories [4]. LV late gadolinium enhancement (LGE) of ≥ 1 bull’s eye segment (s) of CE-CMR and family history/genetics were included as the major criteria, and the minor criteria were considered as follows: 1. Global LV systolic dysfunction with or without LV dilatation; 2. Regional LV hypokinesia or akinesia of the LV free wall, septum, or both; 3. Inverted T waves in the left precordial leads (V4-V6); 4. Low QRS voltages in limb leads; 5. Frequent ventricular premature contractions (> 500 per 24 h) and nonsustained or sustained ventricular tachycardia with RBBB morphology; 6. Family history and(or) genetics.

### Echocardiography

Echocardiographic measurements were performed by a board-certified echocardiographer. Two-dimensional biographic imaging of all subjects was performed using GE Vivid E95 equipment (Norway) with a 2.5 MHz transducer. All images were stored in a digital format for offline analysis (EchoPAC version 113; GE Healthcare). Dynamic 2D images of the apical four- and two-chamber views were used to analyze the auto-EF, and three anchor points were set within the LV cavity, including two at the level of the mitral valve annulus and one at the LV apex. Endocardial borders were then detected and traced automatically by the software during the whole heart cycle to calculate left ventricular end-diastolic volume, left ventricular end-systolic volume, and left ventricular ejection fraction (LVEF). When needed, corrections were performed manually.

Left ventricular longitudinal strain was assessed using two-dimensional speckle tracking echocardiography in three standard apical views. The region of interest was adjusted to ensure optimal analysis of most of the LV walls based on the American Society of Echocardiography /European Association of Echocardiography consensus statement [6]. Each apical view assessment produced six segmental values of the peak systolic longitudinal strain. For each segment, the software automatically separated the myocardium into subendocardial and subepicardial layers. The peak systolic longitudinal strain of the subendocardial myocardium and subepicardial myocardium were derived. The “transmural strain gradient” was defined as the absolute difference between the subendocardial and subepicardial myocardium in longitudinal vectors, and the “transmural strain gradient ratio” was defined as the transmural strain gradient divided by subepicardial myocardial strain.

### CMR

All participants underwent CMR at 1.5 T or 3 T (Aera and Avanto 1.5 T; Prisma 3 T scanner; Siemens Healthcare, Erlangen, Germany). All images were analyzed using CVI42 software (Circle Cardiovascular Imaging Inc., Calgary, Canada). Measurements were performed by two experienced cardiologists blinded to both clinical and genetic data (E.N. and S.M.F.). LV late gadolinium enhancement (scar) quantification was performed in the short-axis slices using manually drawn endocardial and epicardial borders and a semiautomated 5 SD approach with minimal manual adjustment and was expressed in grams and as a percentage of the total LV mass.

### Gene studies

The chip capture sequencing technique was used to capture all exons and shear sites related to ACM, and high-throughput sequencing was used to test the genetic mutations. Sanger sequencing was performed to verify suspected mutation sites responsible for the disease, which were detected by high-throughput sequencing. All gene assay were performed using Mygenostics.

### Statistics

Statistical analysis was performed using SPSS 25.0 and GraphPad Prism 8.0.1. The family members were divided into three groups based on whether they carried the TMEM43 mutation and had been diagnosed with ALVC. Differences in continuous variables between the two groups were analyzed using Student’s t test.

## Results

### Clinical information

**Fig. 1:**
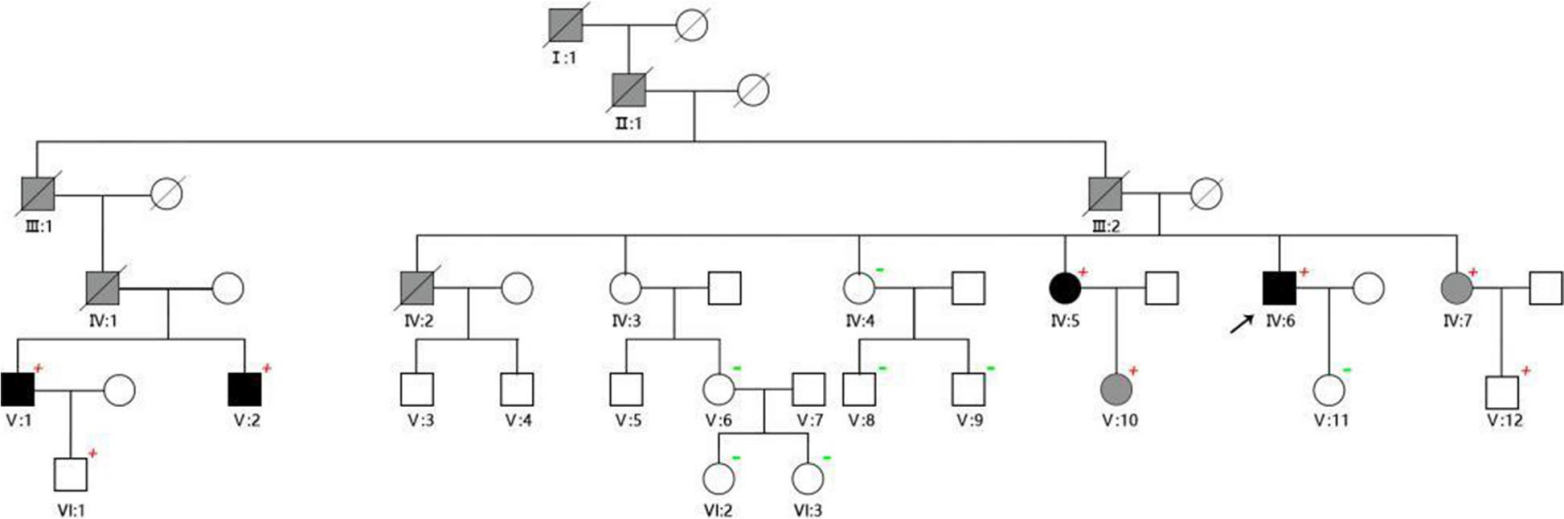
Pedigree of the studied family; Males (squares) and females (circles) diagnosed with ALVC are shown in black. Patients marked in grey refer to suspected ALVC or borderline males/females. Red plus signs represent patients with TMEM43 mutations and green minus signs indicate family members without TMEM43 mutations

Figure 1 shows the pedigree of the ALVC family. In July 2018, the proband (patient IV:6) was admitted to the intensive care unit because of sudden cardiac arrest. After 12 days, he regained consciousness and was transferred to the cardiovascular department and implanted with a cardioverter defibrillator. Fifteen family members were also evaluated.

**Fig. 2:**
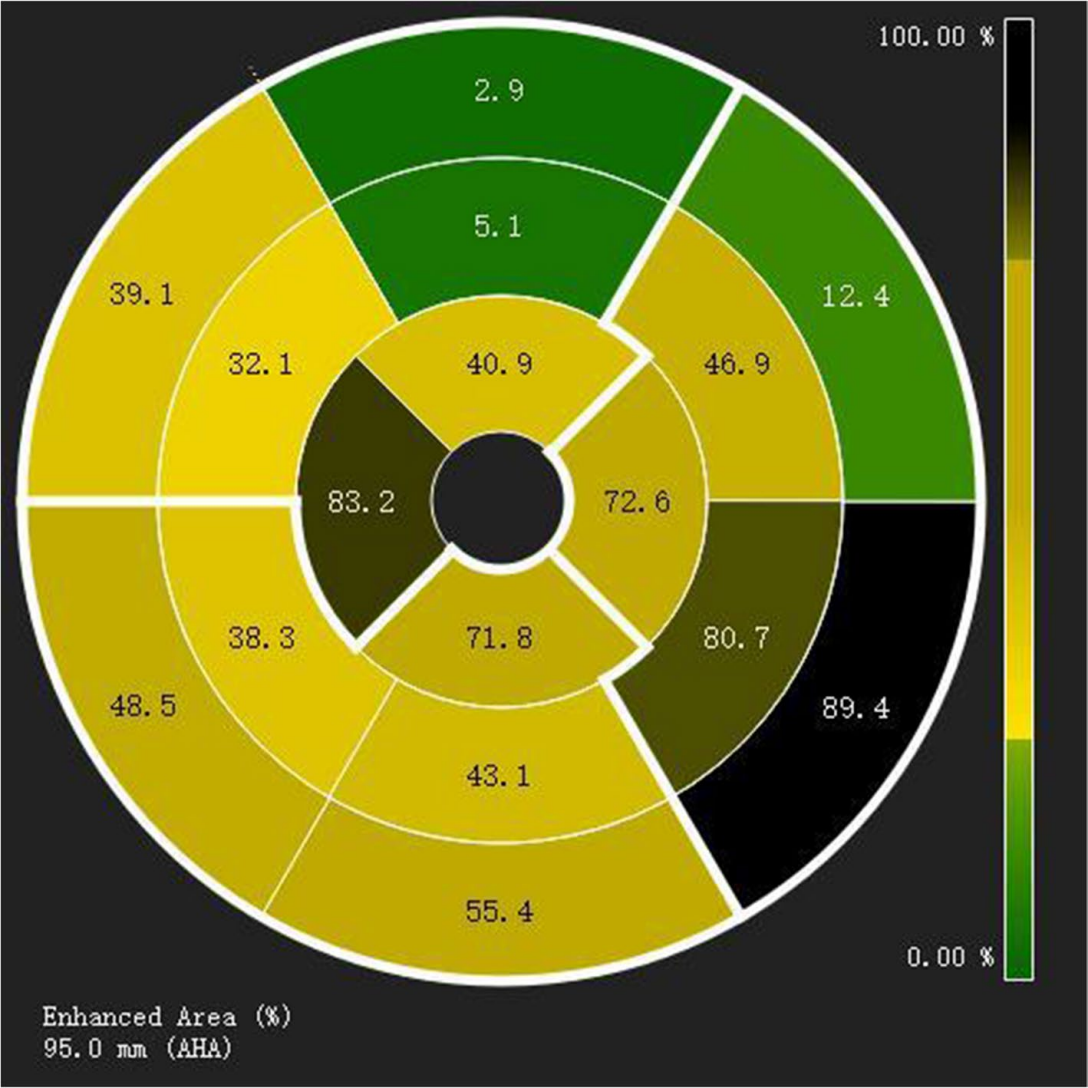
Distribution of left ventricular late gadolinium enhancement of the proband in cardiovascular magnetic resonance bull’s eye view of the 17-segment American Heart Association, which shows evident myocardial scarring in the lateral and posterior LV walls

TMEM43 mutations were detected in eight family members, of which 4 were diagnosed with ALVC according to the Padua criteria (Table 1), which were included in the group with genotype and phenotype positivity (patients IV:5, IV:6, V:1 and V:2). The 4 patients who had TMEM43 mutations and were not diagnosed with ALVC were divided into genotype-positive and phenotype-negative groups (patients IV:7, V:10, V:12 and VI:1). One patient experienced sudden cardiac death, and three experienced dyspnea. Regional left ventricular hypokinesia or akinesia of the left ventricular free wall was evident in four patients, and five had a reduced segmental left ventricular longitudinal strain value in the bull’s eye map. One had a dilated left ventricle. The left ventricular ejection fraction was depressed in 2 family members, and three had a reduced global longitudinal strain. Three patients had low QRS voltage in limb leads. Twenty-four-hour Holter monitoring indicated that five patients had frequent ventricular premature contractions (> 500 per 24 h), and three had nonsustained ventricular tachycardia. Coronary artery disease was excluded by coronary angiography or coronary computed tomographic angiography in all eight patients with TMEM43 mutations. Two-dimensional echocardiography revealed normal right ventricular function and strain. Late gadolinium enhancement was detected in three patients (Fig. 2). Male patients showed more severe clinical features and were diagnosed with ALVC at an earlier age than women.

**Table 1:** Clinical diagnostic features in genetically affected individuals

ID	IV:5	IV:6	V:1	V:2	IV:7	V:10	V:12	VI:1
Age	53	51	48	43	48	29	18	24
Sex	F	M	M	M	F	F	M	M
LVEDVI(ml/m^2^)	55.9	78.5	61.5	44.1	43.4	29.2	42.3	45.2
LVEF (%)	60	40	48	59	62	62	62	62
LV regional wall motion abnormality	Posterior and lateral wall	Posterior and lateral wall	Posterior and lateral wall	Posterior wall	(-)	(-)	(-)	(-)
Reduction in segmental longitudinal strain	Posterior and lateral segments	Posterior, lateral and anterior segments	Posterior and lateral segments	Posterior and lateral segments	Lateral and septal segments	(-)	(-)	(-)
GLS	-20.1%	-13.75%	-14.1%	-17.9%	-23%	-23%	-22.7%	-18.5%
TMEM43	( +)	( +)	( +)	( +)	( +)	( +)	( +)	( +)
ECG	Low QRS voltages in limb leads	Low QRS voltages in limb leads	Low QRS voltages in limb leads, V4-V6 inverted/ flattened T waves	(-)	(-)	(-)	(-)	(-)
Holter	Ventricular premature contractions: 1075 /24 h	Ventricular premature contractions: 15,367 /24 h, VT ( +)	Ventricular premature contractions:1075 /24 h, VT ( +)	Ventricular premature contractions: 1516 /24 h, VT ( +)	Ventricular premature contractions: 824 /24 h	Ventricular premature contractions: 3 /24 h	Ventricular premature contractions: 4 /24 h	Ventricular premature contractions: 4 /24 h
LGE	(-)	( +)	( +)	( +)	(-)	(-)	(-)	(-)
Diagnostic Criteria	1 M + 3 m	2 M + 3 m	2 M + 4 m	2 M + 2 m	1 M + 1 m	1 M	1 M	1 M

*ID* individual number on pedigree (Fig. 1), *M* male, *F* female, *LVEDVI* left ventricular end-diastolic volume index, *LVEF* left ventricular ejection fraction, *LV* left ventricle, *GLS* global longitudinal strain, *ECG* electrocardiogram, *CMR* cardiovascular magnetic resonance, *LGE* late gadolinium enhancement, *M* major criteria, *m* minor criteria

Padua criteria: Major criteria include left ventricular late gadolinium enhancement (LGE) of ≥ 1 bull’s eye segment (s) of CE-CMR and family history/genetics. Minor criteria include global LV systolic dysfunction with or without LV dilatation, regional LV hypokinesia or akinesia of the LV free wall, septum, or both, inverted T waves in the left precordial leads (V4-V6), low QRS voltages in limb leads, frequent ventricular premature contractions (> 500 per 24 h), nonsustained or sustained ventricular tachycardia with RBBB morphology; and family history and/or genetics. Diagnosis is fulfilled by 2 major or 1 major and 2 minor criteria from different categories.

### Echocardiographic parameters and 24-h Holter monitoring in different groups

**Fig. 3:**
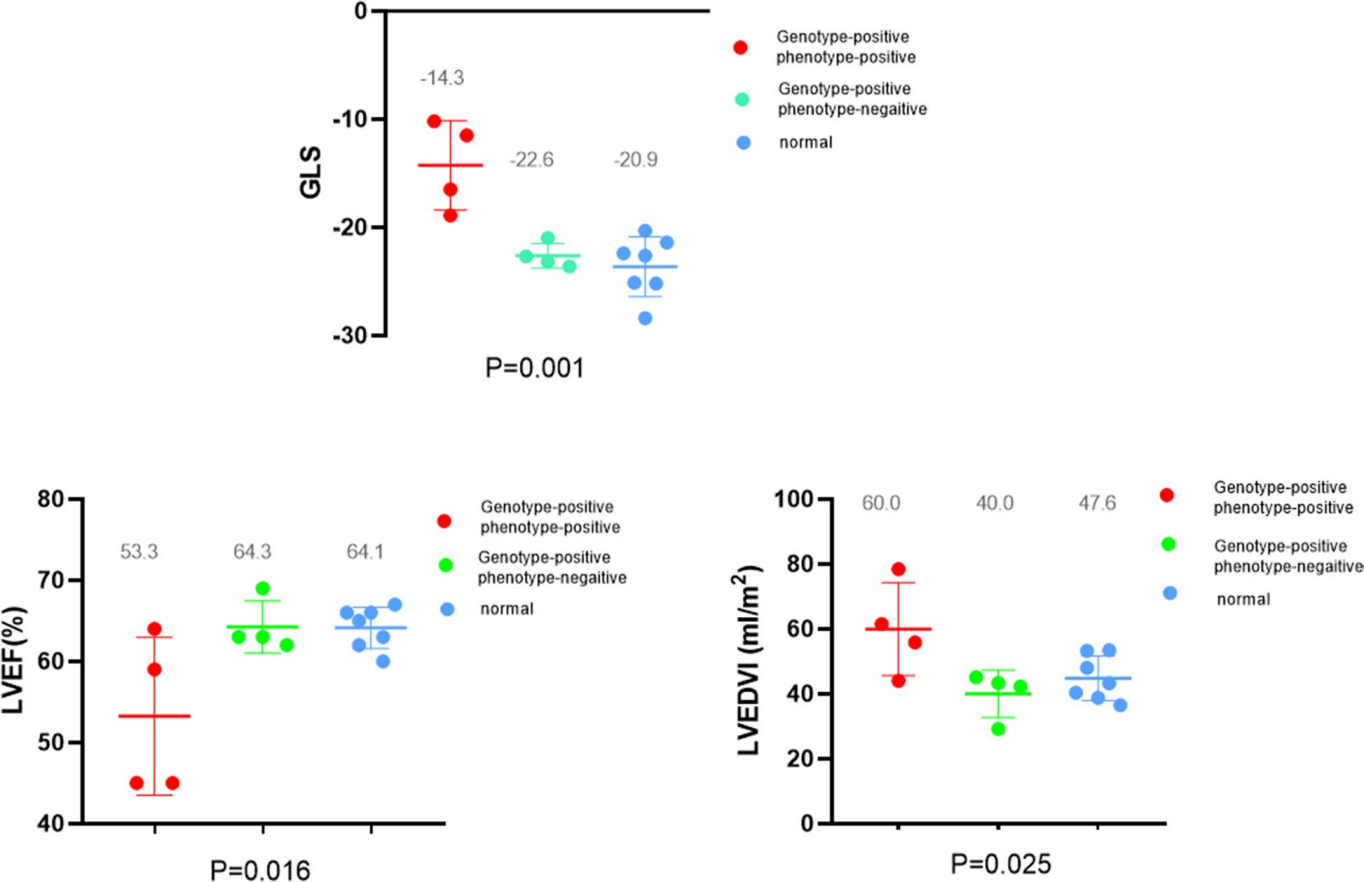
Echocardiographic parameters in different groups. (A) Global longitudinal strain; (B) left ventricular ejection fraction; (C) left ventricular end-diastolic volume index

**Fig. 4:**
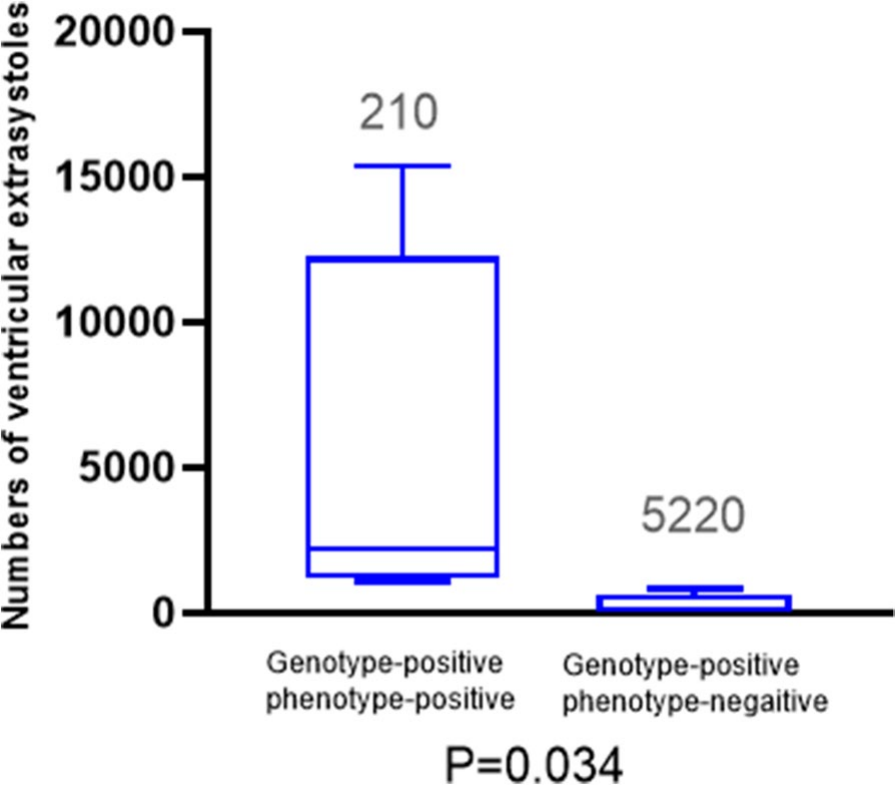
Numbers of ventricular premature contractions in24-hour Holter monitoring in different groups

Global longitudinal strain and left ventricular ejection fraction were reduced, while the number of ventricular premature contractions in 24 h and LVEDVI were increased in patients diagnosed with ALVC. Global longitudinal strain and LVEF in genotype-positive and phenotype-negative patients were not significantly reduced compared to those in healthy individuals (Figs. 3 and 4).

### Left ventricular layer-specific strain

**Fig. 5:**
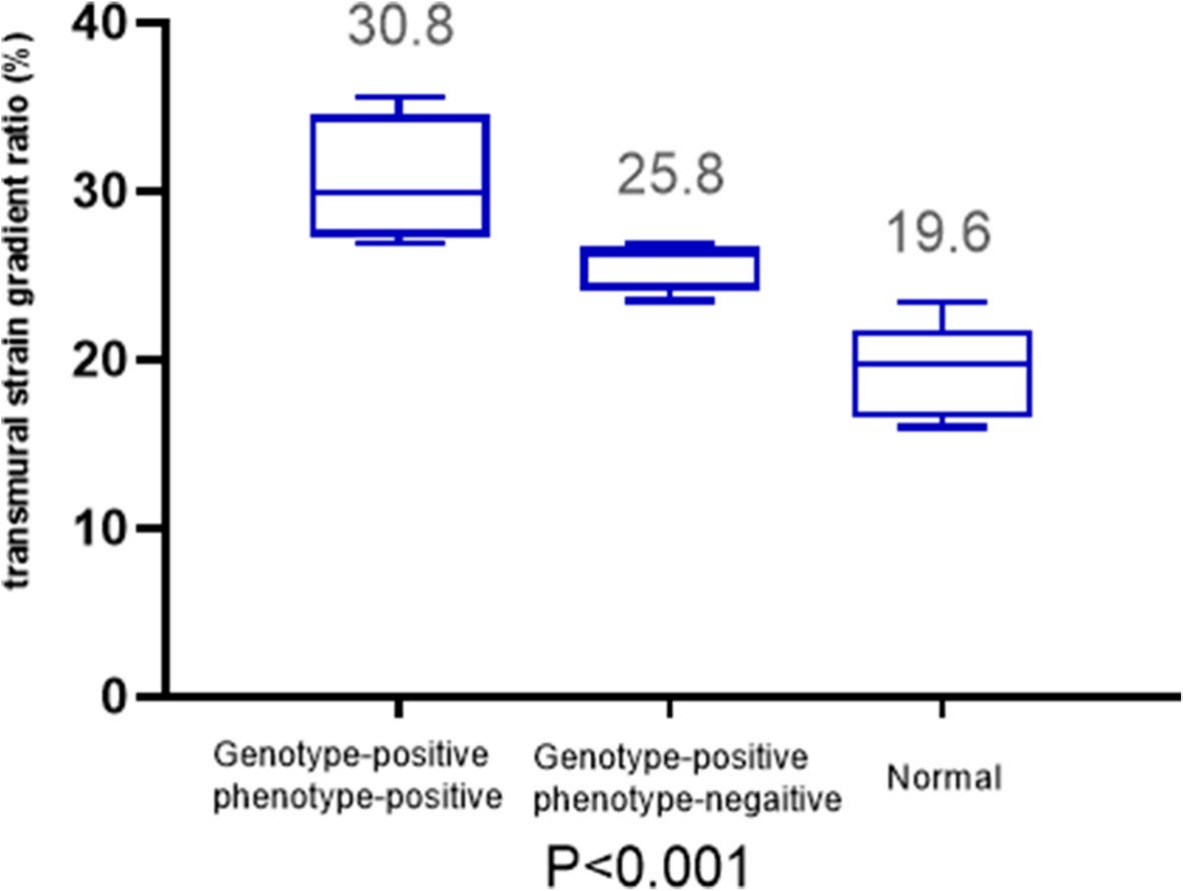
Transmural strain gradient ratio in genotype-positive and phenotype-positive patients, genotype-positive and phenotype-negative patients, and normal members in this family

**Fig. 6:**
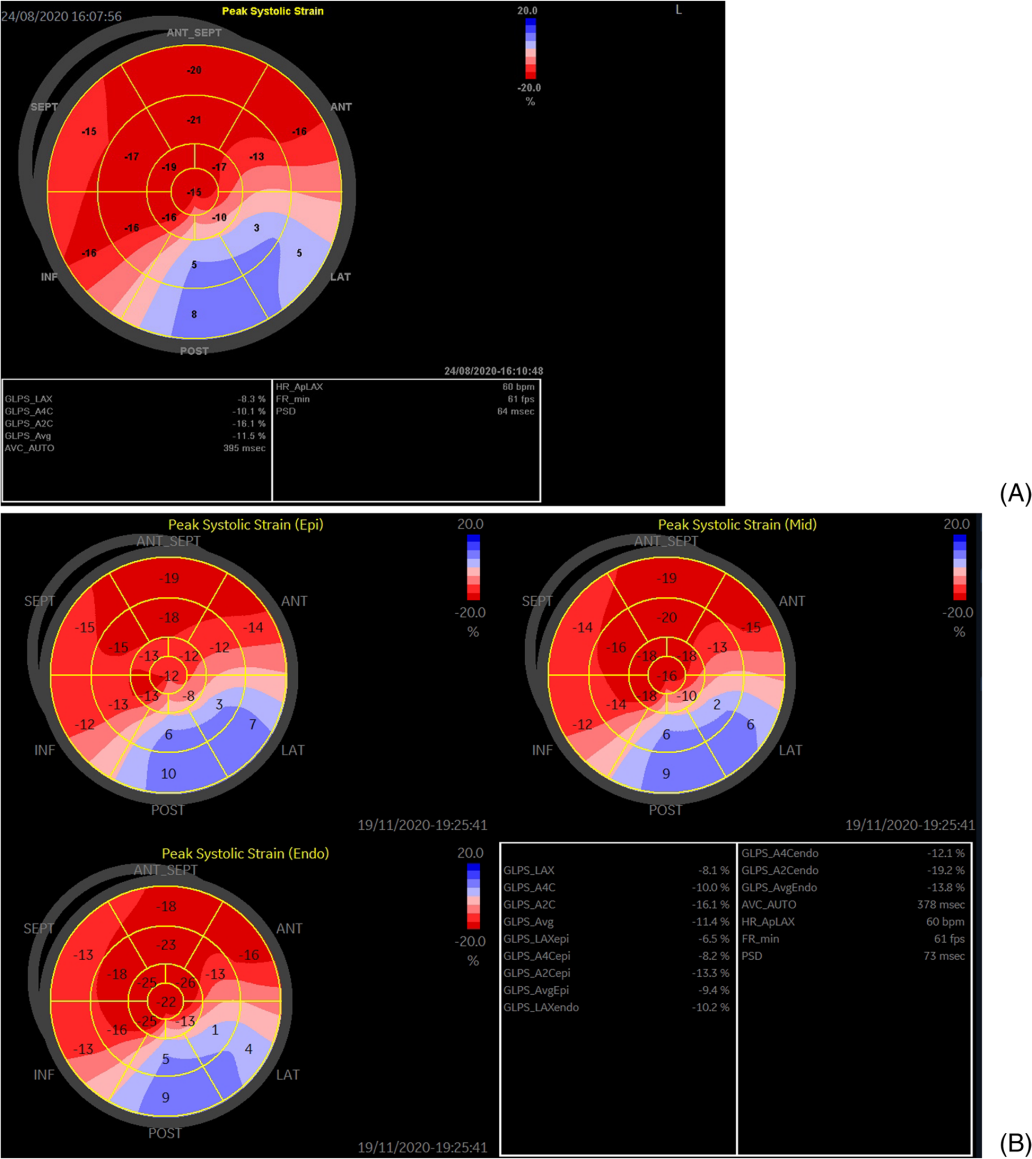
(A) Left ventricular longitudinal strain of the proband. (B) Left ventricular layer-specific strain of the proband

The left ventricular layer-specific strain showed that the transmural strain gradient ratio was increased in patients diagnosed with ALVC, and it was elevated in the genotype-positive and phenotype-negative groups compared with that in healthy individuals (Figs. 5 and 6).

## Discussion

Arrhythmogenic cardiomyopathy is an inherited cardiac disease characterized by fibrofatty replacement of the myocardium, in both the right and left ventricles [1]. We report a family with arrhythmogenic left ventricular cardiomyopathy that included 15 family members. TMEM43 mutations were evident in eight patients, and four were diagnosed with ALVC according to the Padua criteria [4].

Mutant TMEM43 was first described in families from Newfoundland (Canada) in 2008, as the cause of a fully penetrant aggressive disease with a high incidence of malignant ventricular arrhythmias, and was defined as ACM/ARVC type 5 [2]. Previous studies reported that one of the clinical characteristics of the mutation is dilated left ventricle [7]. However, in our report, the left ventricular volume index increased mildly only in the proband. Male patients showed more severe clinical features and were diagnosed with ALVC at an earlier age than women, which is consistent with prior studies [4, 7].

Inverted T waves in left precordial leads, low QRS voltages in limb leads, frequent ventricular premature contractions, and nonsustained or sustained ventricular tachycardia were ECG depolarization, repolarization abnormalities, and ventricular arrhythmias in the Padua criteria [4]. In the current report, three patients had low QRS voltages in limb leads, frequent ventricular premature contractions were detected in 5 patients, and the number of ventricular premature contractions was higher in the genotype-positive group diagnosed with ALVC than in the other groups. Three patients had nonsustained ventricular tachycardia in the current report.

Five family members underwent CMR, and LGE was evident in three patients, suggesting cardiac scarring. Fibrofatty myocardial replacement was confirmed by endomyocardial biopsy in prior studies [4, 8]. Previous studies indicated that half of the ALVCs with LV LGE had no wall abnormalities, and LV fibrosis may deteriorate left ventricular systolic function and lead to a higher prevalence of subepicardial fibrosis [8, 9].

Left ventricular ejection fraction was reduced in two patients, while GLS was decreased in all 4 patients who were diagnosed with ALVC according to the Padua criteria, suggesting that left ventricular strain better evaluates left ventricular function than left ventricular ejection fraction. Region left ventricular akinesia or dyskinesia was evident in five patients, and it was more apparent in the posterior and lateral LV walls. The reduction of the segmental left ventricular longitudinal strain in bull’s eye map was similar to that of two-dimensional echocardiography, which showed that left ventricular strain values were more frequently reduced in the posterior and lateral left ventricular walls. Regional left ventricular hypokinesia or akinesia was one of the diagnostic criteria for ALVC; however, the reduction in left ventricular strain in the bull’s eye segments was more sensitive and accurate than two-dimensional echocardiography, which should be considered in the future diagnostic criteria for ALVC.

Left ventricular layer-specific strain showed that the transmural strain gradient ratio was increased in patients diagnosed with ALVC, and it was elevated in the group with genotype-positive and phenotype-negative patients compared with that in the normal group. A previous study showed that fibrofatty myocardial replacement affects the subepicardial left ventricle more than other layers [8], which may explain the layer-specific strain results. CMR also confirmed that cardiac scars are more apparent in the subepicardium of left ventricle [8, 9]. The findings of left ventricular layer-specific strain were consistent with the pathological characteristics of ALVC, indicating that it may play an important role in the evaluation of ALVC.

## Conclusion

In this family diagnosed with ALVC, global left ventricular longitudinal strain better evaluated left ventricular function than left ventricular ejection fraction, and the segmental left ventricular strain was more accurate and consistent with 2D echocardiography. The transmural strain gradient ratio was elevated in patients diagnosed with ALVC, suggesting that layer-specific strain is useful for evaluating patients with ALVC.

## Data Availability

The datasets during and/or analysed during the current study available from the corresponding author on reasonable request.
